# Uncertainty analysis and optimization for mild moxibustion

**DOI:** 10.1371/journal.pone.0282355

**Published:** 2023-04-17

**Authors:** Honghua Liu, Zhiliang Huang, Lei Wei, He Huang, Qian Li, Han Peng, Mailan Liu

**Affiliations:** 1 Hunan University of Chinese Medicine, Changsha, PR China; 2 Hunan City University, Yiyang, PR China; 3 Hunan Institute of Science and Technology, Yueyang, PR China; Southwest Jiaotong University, CHINA

## Abstract

During mild moxibustion treatment, uncertainties are involved in the operating parameters, such as the moxa-burning temperature, the moxa stick sizes, the stick-to-skin distance, and the skin moisture content. It results in fluctuations in skin surface temperature during mild moxibustion. Existing mild moxibustion treatments almost ignore the uncertainty of operating parameters. The uncertainties lead to excessive skin surface temperature causing intense pain, or over-low temperature reducing efficacy. Therefore, the interval model was employed to measure the uncertainty of the operation parameters in mild moxibustion, and the uncertainty optimization design was performed for the operation parameters. It aimed to provide the maximum thermal penetration of mild moxibustion to enhance efficacy while meeting the surface temperature requirements. The interval uncertainty optimization can fully consider the operating parameter uncertainties to ensure optimal thermal penetration and avoid patient discomfort caused by excessive skin surface temperature. To reduce the computational burden of the optimization solution, a high-precision surrogate model was established through a radial basis neural network (RBNN), and a nonlinear interval model for mild moxibustion treatment was formulated. By introducing the reliability-based possibility degree of interval (RPDI), the interval uncertainty optimization was transformed into a deterministic optimization problem, solved by the genetic algorithm. The results showed that this method could significantly improve the thermal penetration of mild moxibustion while meeting the skin surface temperature requirements, thereby enhancing efficacy.

## 1. Introduction

Moxibustion is a clinical treatment method using an ignited moxibustion cone (or grass) as the heat source to burn or warm the affected area. The moxibustion efficacy mainly comes from the combined effect of thermal effects, non-thermal radiation effects and pharmacological effects [1, 2]. The moxibustion can inhibit the growth of tumours and be beneficial to the growth of tumour-infiltrating lymphocytes [3–5]. The moxibustion showed desirable merits in managing menstrual pain [6]. Moxibustion can improve pain, function and quality of life in KOA patients [7]. The moxibustion uses burning moxa sticks to heat acupoints on the body surface of patients to achieve curative effect [8]. Therefore, the mild moxibustion is essentially a warm stimulation. By stimulating skin receptors, it promotes and regulates the function of the nervous system [9].

Some scholars have studied the distribution of acupoint temperature after moxibustion. The temperature distribution model of moxibustion was established by magnetic resonance imaging, and the temperature distribution under the skin was analyzed by S Nakamura et al. [10]. J. Ying et al. studied the therapeutic effects of varying moxibustion methods by observing the effects of different moxibustion methods such as ginger separated moxibustion, suspension moxibustion and substance separated moxibustion on the local temperature of the points in the Zusanli point [11]. L. Ying et al. studied the temperature field simulation of isolated pig abdominal skin tissue [12].

Existing mild moxibustion studies consider the operating parameters as deterministic parameters to investigate their influences on efficacy. However, the moxa stick manufacturing errors, the moxibustion environment and the object of operation inevitably bring fluctuations to the operating parameters in actual treatments. If this effect is considered, the moxibustion parameters should be regarded as uncertain. In the moxibustion parameter optimization, the unreliable optimal solution will be obtained without considering the parameter uncertainties. It may cause excessive skin surface temperature to burn the skin or over-low temperature to reduce efficacy. Therefore, in the moxibustion parameter design, parameter uncertainty cannot be ignored. To this end, this paper proposes an efficient interval uncertainty optimization method to deal with the uncertainties in the moxibustion parameter optimization. As a new optimization method, interval optimization fully considers the fluctuation range of operating parameters [13–15], attracting increasing attention in mechanics, acoustics, and heat transfer [16–25]. The interval uncertainty optimization can fully consider the influence of the parameter uncertainties, ensuring that the obtained optimal solution meets the reliability requirements. The moxibustion parameters generally include the moxa-burning temperature, moxa stick sizes, stick-to-skin distance and skin moisture content. In this paper, the interval uncertainty optimization method is adopted, the uncertainty is measured as an interval model, and an optimization model of mild moxibustion parameters is established. In this paper, the interval uncertainty optimization method was adopted, which measures the uncertainties as interval models. Thus, the moxibustion parameter optimization model was established, which was solved to obtain the optimal solution meeting the reliability requirements.

This paper analyzed the influence of the mild moxibustion parameter uncertainties on the efficacy and optimized the parameters, which can meet the reliability requirements and achieve optimal efficacy. Firstly, the mild moxibustion simulation model was established, and the corresponding indicator parameters were defined. Secondly, a single factor analysis was conducted after setting the operating parameters, and the constraint and objective functions were created, based on a surrogate model, using the RBNN regression method. Thirdly, an uncertainty analysis for the treatment procedure was performed to verify reliability under initial conditions. Fourthly, a nonlinear interval optimization for the moxibustion parameters was implemented to improve heat penetration and reliability. The uncertain optimization was solved by nonlinear interval programming and transformed into a deterministic optimization problem. Finally, the deterministic optimization was solved by the genetic algorithm to obtain the optimal solution.

## 2. Methods

### 2.1. Determination of mild moxibustion indicators

#### 2.1.1. Mild moxibustion simulation model

In this paper, we construct a multilayer physical model in COMSOL to simulate the biological skin tissue. The biological tissues in the proposed simulation model are defined as three layers, including the skin, fat and muscle layers. The thicknesses of the three tissue layers are set to 2.2 mm, 12.4 mm and 10.4 mm.

During mild moxibustion, not only the operating parameter uncertainties of the moxa stick affect the mild moxibustion effectiveness, but also the skin moisture content with age leads to the fluctuation of skin thermophysical parameters. The variation can be approximated as.


$$\rho (\omega_{c})=1.3-0.3\omega_{c}$$ (1)

$$c(\omega_{c})=4.19(0.37+0.67\frac{\omega_{c}}{\rho })$$ (2)

$$k(\omega_{c})=4.19(0.133+1.36\frac{\omega_{c}}{\rho })$$ (3)

The thermal and physical parameters of skin tissue vary with water content *ω*_*c*_. The parameters include the density *ρ*, constant pressure heat capacity *c*, and thermal conductivity *k*.

The biological tissue characteristic parameters of the skin are listed in Table 1.

**Table 1:** The biological tissue characteristic parameters.

Biological tissue	Constant pressure heat capacity *C* (J∙ kg^–1^∙ K^–1^)	Density *ρ*(kg∙ m^–3^)	Thermal conductivity *k* (W∙ m^–1^∙ K^–1^)
fat	2348	911	0.21
muscle	3421	1090	0.49

**Fig 1:**
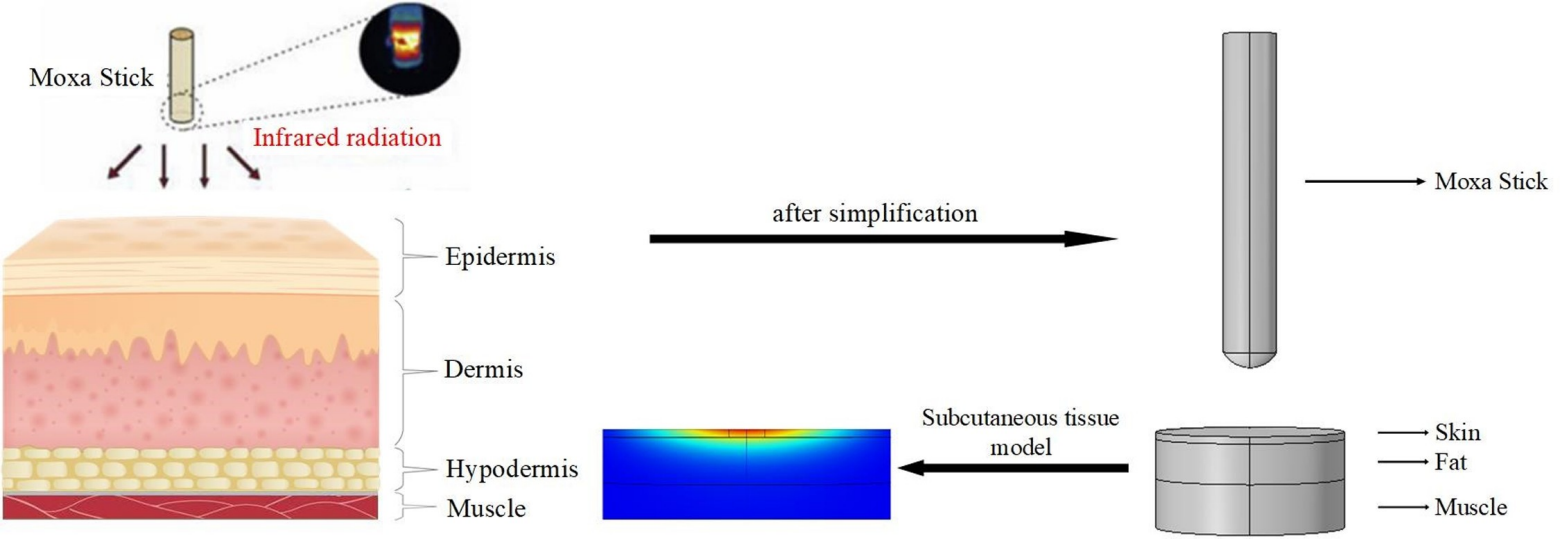
COMSOL simulation model.

As shown in Fig 1, the biological tissue model is established in COMSOL. The head of the moxa stick is a burning hemisphere, and the combustion heat transfer mode is mainly thermal radiation. The following thermal convection is not considered: the head of the moxa stick is a high-temperature heat source, and the thermal convection between it and the air is ignored. The heat conduction caused by the head of the moxa stick to the tail of the hand-held moxa stick is neglected, so the heat convection between the rear of the hand-held moxa stick and the contact air is ignored. The simulation model of combustion heat radiation of moxa sticks is established.

The temperature distribution of subcutaneous tissue during moxibustion treatment is showed in Fig 1. The mesh division of skin tissue near the moxibustion head area is more detailed during modelling.

#### 2.1.2. Mild moxibustion indicators and mathematical model

To calculate the temperature distribution in the skin tissue, the heat source in the heat transfer process needs to be determined. The heat source is gained from the moxa stick burning end through the radiative heat transfer. The temperature distribution of the treatment area was obtained by performing biological heat transfer calculations on the skin tissue.

The relationship between the moxa stick combustion and skin surface thermal radiation is:

$$\begin{array}{l} J=\varepsilon e_{b}\left(T\right)+\rho_{d}G\\ \;e_{b}\left(T\right)=n^{2}\delta T^{4} \end{array}$$ (4)

where *J* denotes the effective radiation, *G* represents the input radiation, *e*_*b*_(*T*) is the radiative heat flux density of the blackbody as a function of temperature, *ρ*_*d*_ is the surface reflectance, *T* denotes the temperature, *σ* is the blackbody radiation constant (i.e., 5.67 × 10^-8^W∙ m^-2^∙ K^-4^), *n* is the refractive index of the transparent medium (*n* = 1).

The Pennis equation is used to solve the treatment [26]:

$$\rho c\frac{\partial T}{\partial t}=\nabla \cdot \left(k\nabla T\right)+\omega_{b}C_{b}\left(T_{b}-T\right)+q_{m}+q_{r}$$ (5)

where *T*, *ρ*, *c* and *k* denote the temperature, density, specific heat, and thermal conductivity of the tissue, respectively. *ω_b_* represents the blood perfusion rate, *C*_*b*_ and *T*_*b*_ are the specific heat and temperature of the blood, *q*_*m*_ is the heat production rate of the tissue metabolic, *q*_*r*_ denotes the external heat source.

In mild moxibustion, the high temperature can generally lead to excellent efficacy. But the over-high temperature will make the patient uncomfortable and even burn the patient. According to clinical experience, the skin surface temperature higher than 45.5℃ during moxibustion makes patients feel uncomfortable. Also, the thermal penetration is a mild moxibustion indicator, i.e., the tissue temperature at 5mm from the skin surface. This indicator should be increased to maximize the efficacy of mild moxibustion. Thus, the skin surface temperature *ST* and thermal penetration *HPM* were considered indicators to measure the mild moxibustion efficacy.

### 2.2. Single factor analysis of operating parameters

Univariate analysis is the analysis of only one variable, and only one direction of experimental treatment. It is necessary to determine the relationship between various operating factors and the efficacy during mild moxibustion. The univariate analysis is to change a certain working parameter while keeping the other operating parameters unchanged. Through simulation and experimental treatment, the influence of this factor on skin temperature distribution is analyzed.

Under an ideal condition, the temperature distribution of mild moxibustion takes the moxibustion point as the center, and the temperature decreases along the radial direction. In this section, univariate analysis experiments were conducted to analyze the influence of each factor on the heat penetrating *HPM* and the skin surface temperature *ST* of mild moxibustion. The findings are expected to help doctors determine the parameters of moxibustion faster and better in clinical practice.

#### 2.2.1. Mild moxibustion working parameter setting

**Fig 2:**
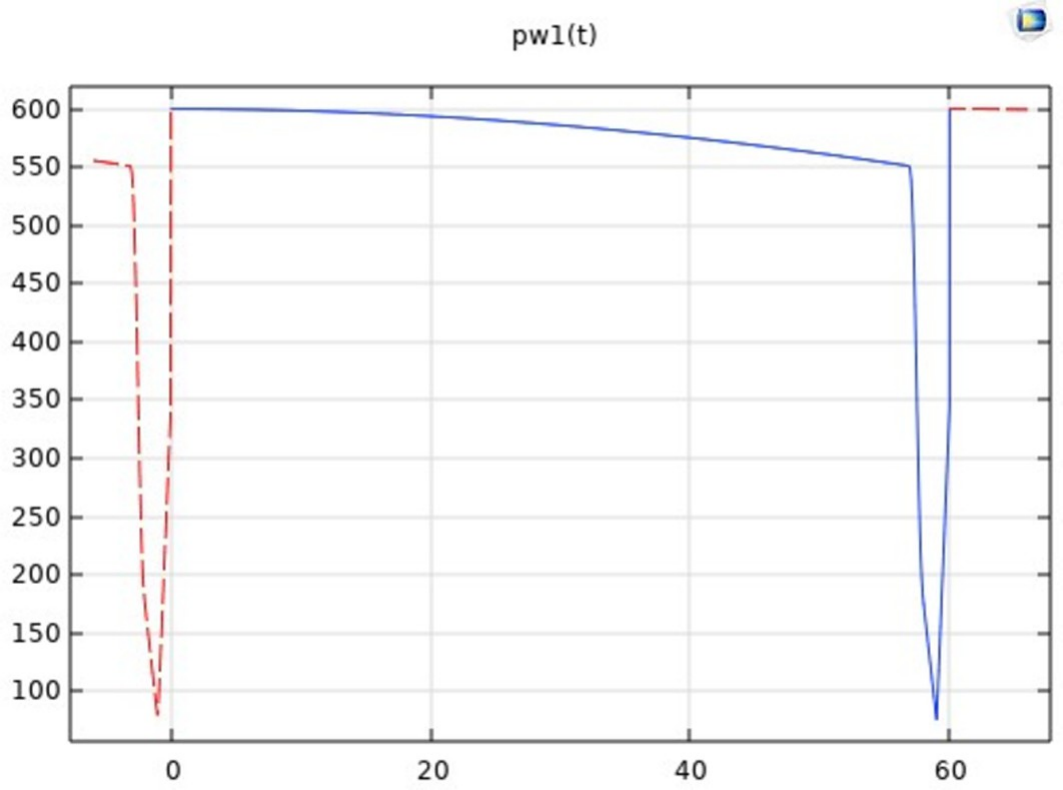
Temperature curve of wormwood.

The maximum combustion temperature of moxa sticks is different with specifications and manufacturers, but the temperature variation law is similar. The moxa sticks are lit, then they burn and reach the highest temperature. With the progress of combustion, the ash of sticks will gradually gather at the combustion site, resulting in the gradual reduction of the moxa stick temperature. Therefore, it is necessary to remove the dust on the top of the moxa stick regularly. The ash cleaning is set once a minute to prevent the burning temperature of moxa sticks from decreasing to the point that moxibustion effect cannot be achieved. Since the air has been heated when the moxa stick is removed, the radiation source temperature should not be set to 0°C, but 50°C. The simple harmonics are used to simulate the temperature change at the ash gathering when the moxa sticks burning. The temperature plunges to 50°C during the ash cleaning operation and returns to the maximum temperature when ash cleaning is completed. The mathematical model is shown as Eq (6). The change curve of temperature is shown in Fig 2.


$$T_{a}=400+200\cos(\frac{2\pi }{500}t)$$ (6)

Where *T*_*a*_ is the moxa burning temperature (°C) and *t* denotes time (s).

#### 2.2.2. Mild moxibustion parameter level setting

During mild moxibustion, the-moxa-burning temperature, moxa stick sizes, stick-to-skin distance and skin moisture content are the main parameters affecting the skin temperature distribution. In addition to the operating parameters of moxa sticks, different patients will also produce different efficacy. The difference in skin moisture content leads to different skin thermal properties. Table 2 shows the values of the parameters for each level.

**Table 2:** Parameter values at each level.

Levels	maximum burning temperature of the moxa stick (°C)	moxa radius(mm)	the stick-to-skin distance(mm)	skin moisture content (%)
1	600	12	25	29
2	625	15	30	40
3	650	18	35	52

The skin thermal physical parameters are calculated based on the moisture content. Table 3 lists the values of skin thermal physical parameters under various moisture contents.

**Table 3:** Values of skin thermal physical parameters.

Skin water content (%)	Constant pressure heat capacity *C* (J∙ kg^–1^∙ K^–1^)	Density *ρ* (kg∙ m^–3^)	Thermal conductivity *k* (W∙ m^–1^∙ K^–1^)
29	2222	1213	0.19
40	2502	1180	0.25
52	2826	1144	0.32

#### 2.2.3. Start the single factor analysis

After completing the mild moxibustion simulation model setting, the mild moxibustion working parameters setting and the mild moxibustion working parameters level setting, the study started the single factor analysis.

### 2.3. Establishment of a surrogate model for the mild moxibustion

The efficacy indicators of mild moxibustion mainly include the skin surface temperature *ST* and thermal penetration *HPM*. The moxa-burning temperature, moxa stick size, stick-to-skin distance, and skin moisture content were selected as optimization parameters to study their influences on the moxibustion efficacy.

#### 2.3.1. Optimal Latin hypercube sampling

**Fig 3:**
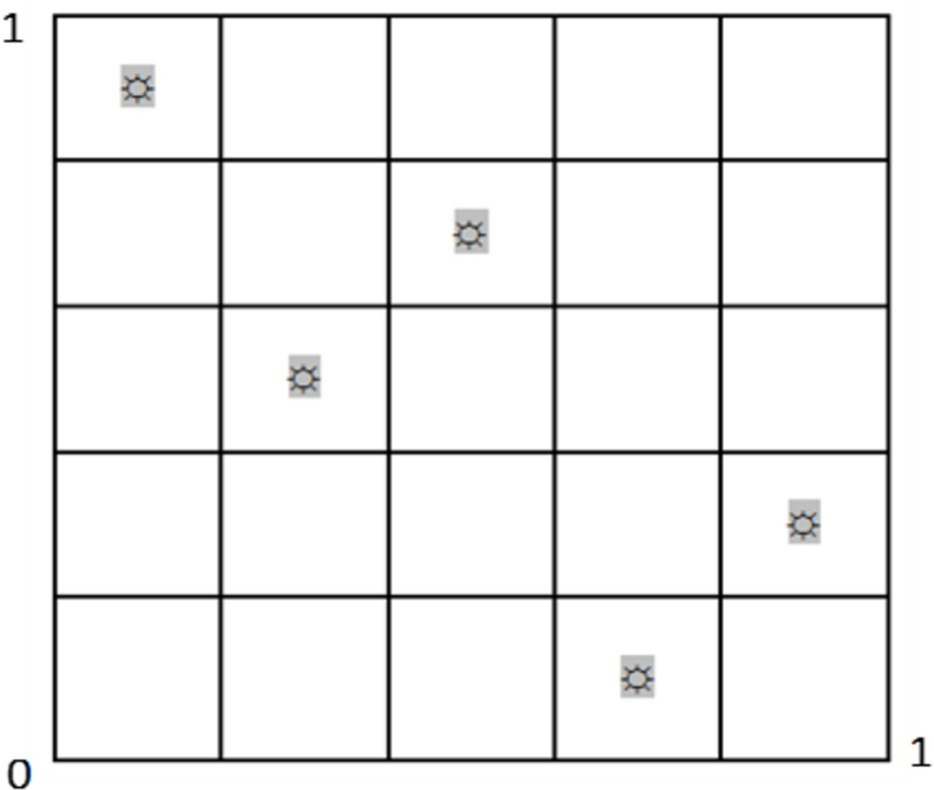
The Latin hypercube sampling.

The interval uncertainty optimization aims to search for an optimal design that satisfies reliability requirements and minimizes (or maximizes) the objective function value. Due to the time-consuming finite element analysis, the optimization solution directly calling the finite element model will lead to high computational costs. About surrogate model functions, such as polynomial response surface (PRS), Kriging (KRG), support vector regression (SVR), radial basis function (RBF), and radial basis neural network (RBNN). Compared with other functions, RBNN has the advantages of high accuracy, simple training, and fast convergence. To improve efficiency, a radial basis neural network (RBNN) concerned with the optimization parameters was established for the constraint and objective functions. As a result, the approximate response functions were provided to solve the interval uncertainty optimization. Establishing RBNN requires experimental design. The Latin hypercube sampling is a widely-used method for multidimensional hierarchical experimental design [27, 28]. The sampling steps are as follows. ① Each space of n-dimensional space is divided into m-number intervals according to equal probability. ② Each interval is randomly sampled once, ensuring that each dimension is studied once. ③ Number of samples are randomly paired to generate an *n* × *m*-type matrix. The result is shown in Fig 3. In this paper, the Latin hypercube is used to sample the design points, which is conducive to the uniform distribution of samples. In this study, 50 experimental design points were obtained using the optimal Latin hypercube sampling method within the design range of operational parameters.

#### 2.3.2 RBNN training

The surrogate model is formulated based on the sampling results to obtain the response surface of the design domain. The RBNN regression method was used to create the surrogate model of the objective function [29, 30]. In the design domain, any design point can be used to verify the accuracy of the surrogate model. To obtain an optimal accuracy surrogate model, an error analysis was performed.

The relative error between the simulation results y(x) and the approximation obtained by the regression function f(x) is written as Eq (7). [31].


$$RE=\frac{y(x)-\overset{⌢}{y}(x)}{y(x)}$$ (7)

The root mean square error (RMSE) is used to evaluate the surrogate model accuracy, expressed as Eqs (8–11).


$$RMSE=\sqrt{\frac{SSE}{k}}$$ (8)

$$R^{2}=1-\frac{SSE}{SST}$$ (9)

$$SSE=\sum_{i=1}^{k}{\left(y_{i}-{\overset{⌢}{y}}_{i}\right)}^{2}$$ (10)

$$SST=\sum_{i=1}^{k}{\left(y_{i}-\bar{y}\right)}^{2}$$ (11)

Here, SSE is the sum of squared errors, k is the number of samples (k = 50), and SST denotes the total sum of squares. *y*_*i*_ is the response value of the simulation model at the i-th sample, ${\hat{y}}_{i}$ is the response value of the surrogate model at the i-th sample, and $\bar{y}$ represents the mean of *y*_*i*_.

#### 2.3.3. Uncertainty analysis of objective function

The mild moxibustion uses the high temperature and smoke generated by burning moxa sticks to penetrate the skin to achieve efficacy. The moxa-burning temperature, the moxa stick size, the stick-to-skin distance and the skin moisture content affect the thermal penetration of mild moxibustion. The moxibustion parameters fluctuate in an interval, resulting in the skin surface temperature and thermal penetration also fluctuating. In the traditional mild moxibustion treatment, the uncertainty is usually ignored. If the uncertainty is ignored, the optimized solution will be unreliable due to the uncertainty of the moxibustion parameters. During the actual treatment, the skin surface temperature may exceed the allowable temperature (45.5°C) and cause skin burns. Therefore, to obtain stable moxibustion efficacy, the uncertainty of moxibustion parameters should be considered to develop uncertain optimization models. The uncertainty of each moxibustion parameter is described as an interval. d represents the designed parameter, the variation range of the parameter can be defined as follow:

$$d\in d^{I}=\left[d^{L},d^{R}\right]$$ (12)

Here, *d*^*I*^ represents the parameter interval, *d^L^* and *d*^*R*^ are the lower and upper bounds of the parameter interval, respectively. The interval is also denoted as Eq (13) [32, 33].


$$\begin{array}{l} d\in d^{I}=\left[d^{L},d^{R}\right]=\{d\left|d^{L}\leq d\leq \right.d^{R},d\in R\}\\ \;=\{d\left|d^{c}-d^{\omega }\leq d\leq \right.d^{c}+d^{\omega },d\in R\}\\ \;\text{=}\langle d^{c},d^{\omega }\rangle ,\;i=1,2,3 \end{array}$$ (13)

Here, *d*^*c*^ and *d*^*ω*^ represent the midpoint and radius of the interval, as Eq (14).


$$d^{c}=\frac{d^{L}\text{+}\;d^{R}}{2}\;,\;d^{\omega }=\frac{d^{R}\text{-}\;d^{L}}{2}$$ (14)

As Eq (11), the interval is determined by the midpoint *d*^*c*^ and the radius *d*^*ω*^. *d*^*c*^ is given as the median value of the parameter. The radius *d*^*ω*^ reflects the variation degree in the parameter, which is usually affected by the burning process of the moxa stick.

**Fig 4:**
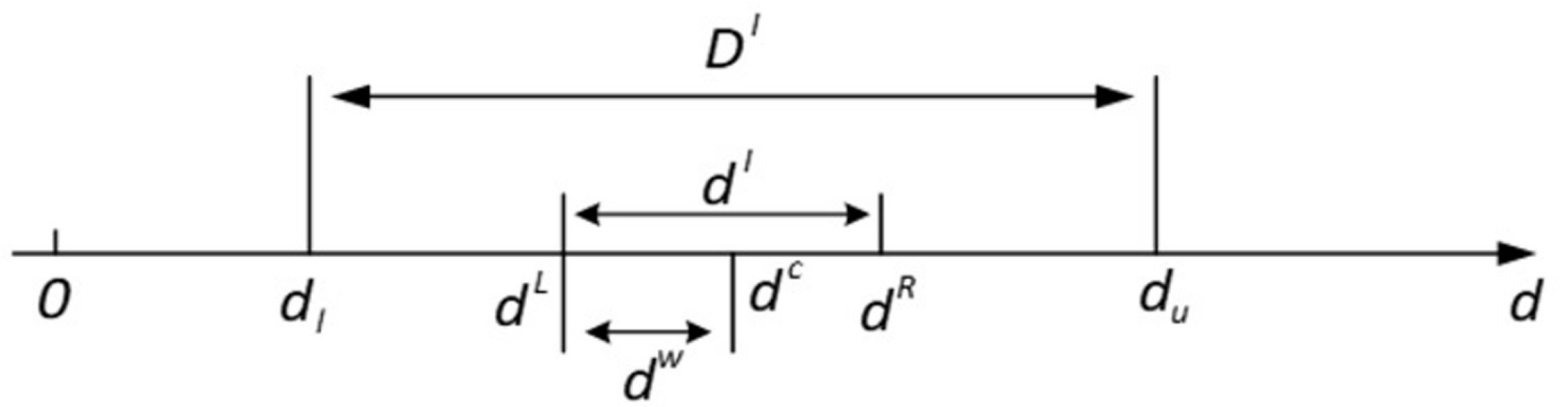
The relationship between interval and design domain.

As shown in Fig 4, *d*^*I*^ should be chosen within a reasonable design domain in the interval uncertainty optimization. The design domain of uncertain parameters is denoted as *D*^*I*^, and its lower and upper bounds are *d*_*l*_ and *d*_*u*_, respectively.

### 2.4. Nonlinear interval optimization model of mild moxibustion

#### 2.4.1. Nonlinear interval optimization modelling

This section presents the interval optimization method for the moxibustion parameters under uncertainties. The purpose is to ensure a skin surface temperature of less than 45.5°C and achieve maximum thermal penetration during mild moxibustion. The interval uncertainty optimization is expressed as Eq (15) [34].


$$\begin{array}{l} \underset{X^{I}}{\min}H\left(X^{I}\right)\\ \;s.t.\\ \;S_{j}\left(X^{I}\right)\leq b_{j}\;,\;j=1,2,\cdot \cdot \cdot ,l\\ \;X_{l}\leq X^{I}\leq X_{u}\\ \;X_{i}{}^{I}=\left[X_{i}{}^{L},X_{i}{}^{R}\right]\;i=1,2,\cdot \cdot \cdot ,n \end{array}$$ (15)

Here, $X^{I}=\left[X_{1}^{I},X_{2}^{I}\cdot \cdot \cdot X_{n}^{I}\right]$ is an n-dimensional interval design vector, *X*_*l*_ and *X*_*u*_ are the lower and upper bounds of the interval variable. *H* and *S* represent the objective and constraint functions, *b*_*j*_ denotes the allowable value of the j-th constraint, l represents the number of constraints. The superscripts I, L and R denote the parameter interval and its lower and upper bounds, respectively. Therefore, the interval uncertainty optimization for the mild moxibustion treatment is to obtain the optimal intervals of moxibustion parameters. By optimizing the moxibustion parameters, the optimal solution of the objective function is achieved while ensuring reliability.

To formulate the interval optimization model for the mild moxibustion treatment, it is necessary to define the objective and constraint functions. In this paper, thermal penetration is defined as the objective function, and the skin surface temperature is considered as the constraint function. Due to the moxibustion parameter uncertainties, the thermal penetration and skin surface temperature are intervals, not fixed values.

The interval design variables are considered as the moxibustion parameters, such as the moxa-burning temperature (*t*_1_), the moxa stick size (*d*), the stick-to-skin distance (*h*),and the skin moisture content(*ω*_*c*_). Changing the moxibustion parameters can optimize the values of the objective and the constraint functions.

Thus, the interval optimization of mild moxibustion treatment is modeled as Eq (16).


$$\begin{array}{l} \underset{d^{c},d^{\omega }}{\max}H\left(d^{I}\right)\\ \;\;s.t.\\ \;\;S\left(d^{I}\right)\leq b\\ \;\;d_{l}\leq d^{I}\leq d_{u}\\ \;\;d_{i}^{I}=\left[d_{i}^{L},d_{i}^{R}\right]\;i=1,2,3 \end{array}$$ (16)

Here, H represents the objective function of thermal penetration, and S denotes the constraint function of skin surface temperature. H and S are the function of moxibustion parameters. $d^{I}\text{=}\left[d_{1}^{I}\;\;d_{2}^{I}\cdot \cdot \cdot d_{n}^{I}\right]$ is a 3-dimensional interval design vector, b represents the allowable value (45.5 °C) of skin surface temperature. *d*_*l*_ and *d*_*u*_ represent the lower and upper bounds of the design domain. $d_{l}^{L}$ and $d_{l}^{R}$ denote the lower and upper bounds of each parameter interval. *d*_*i*_ *i =* 1, 2, 3 are the moxibustion parameter intervals, written as Eq (17).


$$d_{i}^{I}=\langle d_{i}^{c},d_{i}^{\omega }\rangle ,\;i=1,2,3$$ (17)

Therefore, the interval model of mild moxibustion treatment is expressed as Eq (18).


$$\begin{array}{l} \underset{d^{I}}{\max}H\left(\langle d_{i}^{c},d_{i}^{w}\rangle \right)\\ \;\;s.t.\\ \;\;S\left(\langle d_{i}^{c},d_{i}^{w}\rangle \right)\leq b\\ \;\;d_{l}\leq \langle d_{i}^{c},d_{i}^{w}\rangle \leq d_{u} \end{array}$$ (18)

Here, $d^{c}\text{=}\left[d_{1}^{c}\;\;d_{2}^{c}\cdot \cdot \cdot d_{n}^{c}\right]$ is the n-dimensional vector of the interval parameter midpoints. $d^{w}\text{=}\left[d_{1}^{w}\;\;d_{2}^{w}\cdot \cdot \cdot d_{n}^{w}\right]$ is an n-dimensional vector of the interval parameter radius.

This paper introduces the concept of the reliability-based possibility degree of interval (RPDI) when solving the interval optimization model. The purpose is to realize the comparison of different intervals during solution.

For the intervals of *A*^*I*^ and *B*^*I*^, the RPDI is expressed as Eq (19) [35].


$$p_{r}\left(A^{I}\leq B^{I}\right)=\frac{B^{R}-A^{L}}{2A^{\omega }\text{+}2B^{\omega }}$$ (19)

Here, *p*_*r*_ represents the similarity of the intervals. *p*_*r*_ (*A*^*I*^ ≤ *B*^*I*^) has the following features [35]:

① $\text{-}\infty \leq p_{r}\left(A^{I}\leq B^{I}\right)\leq \text{+}\infty ;$
② if $A^{R}\leq B^{L},p_{r}\left(A^{I}\leq B^{I}\right)\geq 1;$
③ if $A^{L}\leq B^{R},p_{r}\left(A^{I}\leq B^{I}\right)\leq 0;$
④ if $p_{r}\left(A^{I}\leq B^{I}\right)\text{=}q,p_{r}\left(B^{I}\leq A^{I}\right)\text{=}1-q$ where $q\in \left(\text{-}\infty ,\text{+}\infty \right)$○

Here, *B*^*I*^ degenerates into an actual number B, and the RPDI can be formulated as Eq (20).


$$p_{r}\left(A^{I}\leq B^{I}\right)\text{=}\frac{B\text{-}A^{L}}{2A^{w}}$$ (20)

#### 2.4.2. Solution of the nonlinear interval optimization

 The constraint function is expressed as Eq (21) [35].


$$S\left(\langle d^{c},d^{\omega }\rangle \right)=\left[S^{L},S^{R}\right]=\left[\underset{d\in \left(d^{c},d^{\omega }\right)}{\min}S\left(d\right),\underset{d\in \left(d^{c},d^{\omega }\right)}{\max}S\left(d\right)\right]$$ (21)

Therefore, the constraint function is transformed into the deterministic constraint as Eq (22).


$$p_{r}\left(S\left(\langle d^{c},d^{\omega }\rangle \right)\leq b\right)=\frac{b-F^{L}}{2S^{\omega }}\geq \lambda$$ (22)

Here, *λ* denotes the RPDI of interval constraint, which is given according to the mild moxibustion treatment requirements. Based on the definition of RPDI, the interval optimization model of mild moxibustion is transformed into the deterministic optimization problem, as Eq (23) [33].


$$\begin{array}{l} \max H\left(\langle d^{c},d^{\omega }\rangle \right)\\ \;s.t.\\ \;p_{r}\left(S\left(\langle d^{c},d^{\omega }\rangle \right)\leq b\right)=\frac{b-F^{L}}{2S^{\omega }}\geq \lambda \\ \;d_{l}\leq d^{c}-d^{\omega }\leq d^{c}+d^{\omega }\leq d_{u} \end{array}$$ (23)

Here, *S*^*L*^ is the lower bound of thermal penetration, and *S*^*w*^ represents its radius. By introducing RPDI, the classical optimization algorithms can be used to solve the uncertain interval optimization. The interval uncertainty optimization of mild moxibustion involves nested optimization. In the outer layer, the genetic algorithm is used to optimize *d*^*c*^. In the inner layer, the sequential quadratic programming is applied to calculate the constraint reliability.

## 3. Result

### 3.1. Single factor analysis of operating parameters

#### 3.1.1. Effect of maximum combustion temperature on temperature distribution of moxa

**Fig 5:**
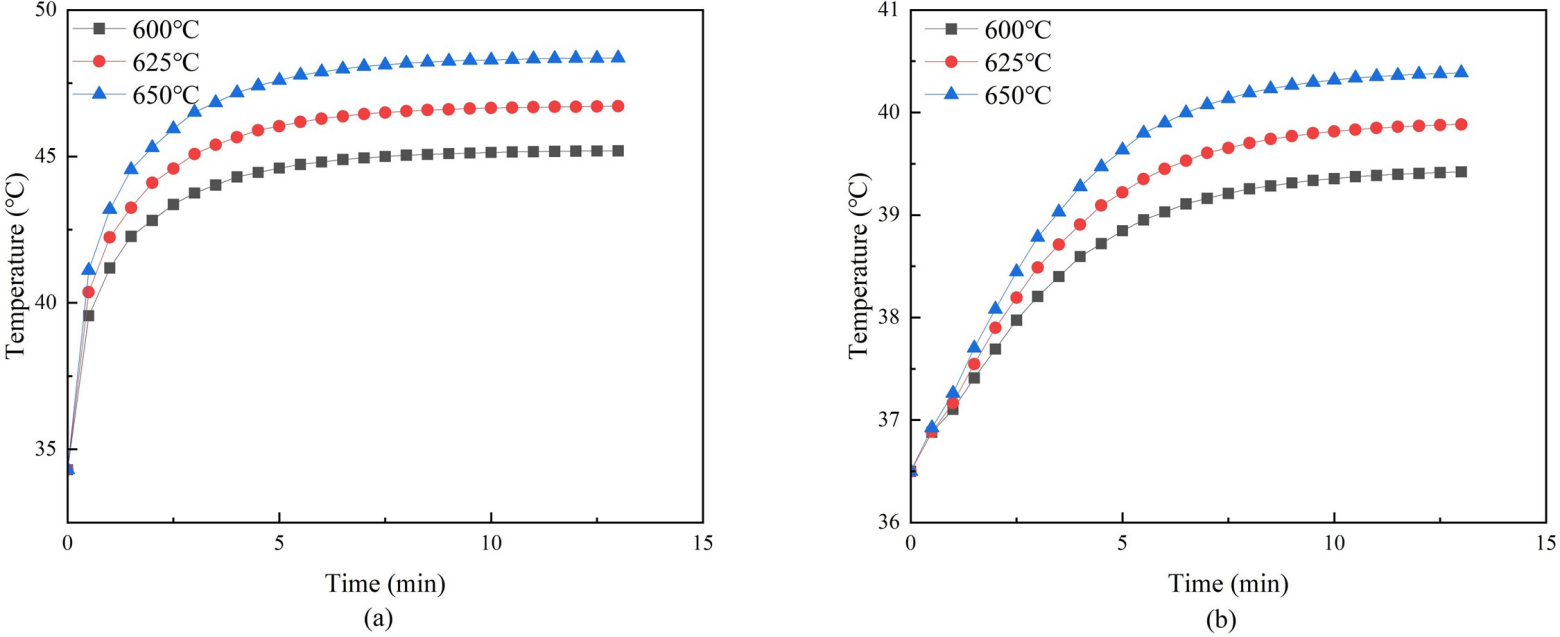
**(a)** Temperature curve of moxibustion point when moxa-burning temperature changes. **(b)** The temperature curve at 5mm under moxibustion point when moxa-burning temperature changes.

**Fig 6:**
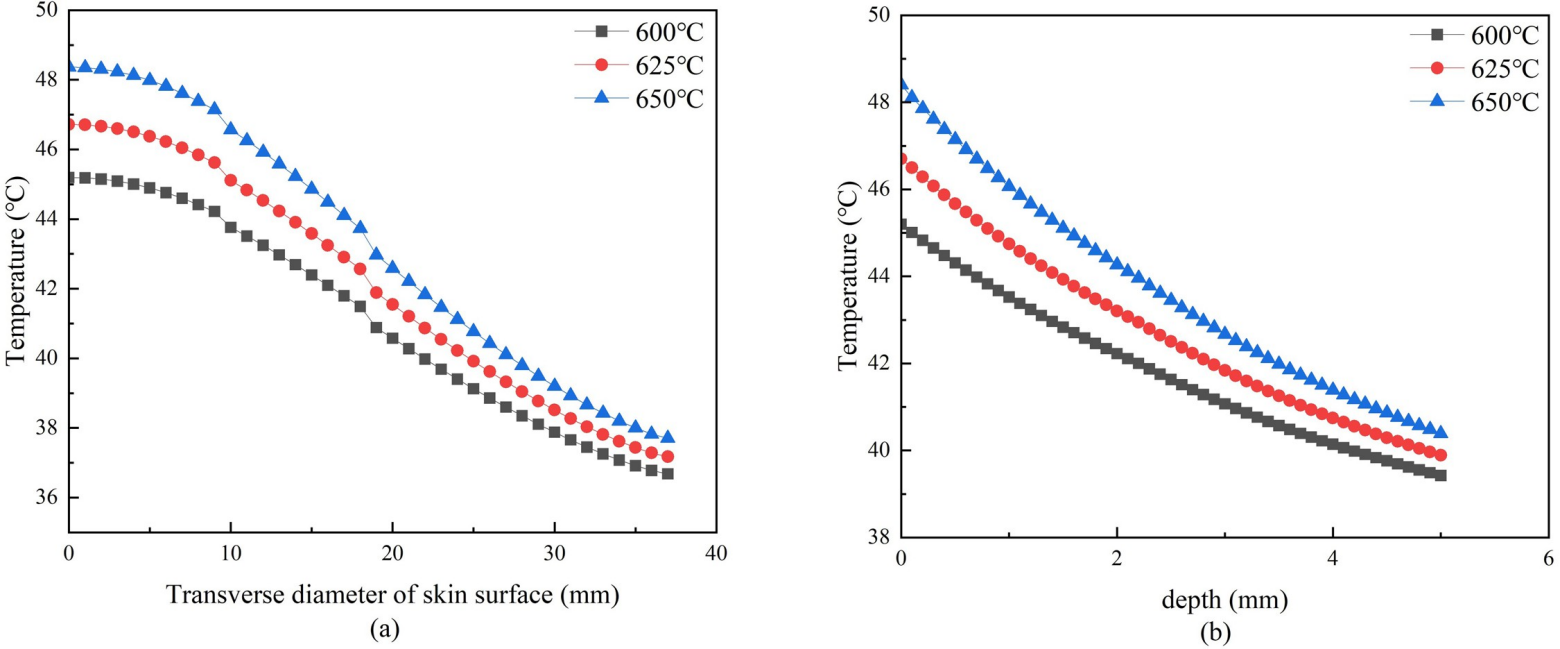
**(a)** Lateral skin temperature curve when moxa-burning temperature changes. **(b)** Vertical skin temperature curve when moxa-burning temperature changes.

Under the conditions that the moxa stick size, stick-to-skin distance, and skin moisture content were fixed, the moxa-burning temperature was varied under the conditions. Three moxa-burning temperature were set to simulate the temperature distribution on and inside the skin after mild moxibustion, as shown in Figs 5 and 6.

Fig 5 shows that the higher the moxa-burning temperature, the higher the skin surface temperature and the temperature at 5mm under the moxibustion point. The temperature of the moxibustion point increased rapidly in the first five minutes. After about 5 minutes, the skin surface temperature of the moxibustion point was gradually stable, and the temperature rise rate was reduced. Fig 6 showed that the slope of the temperature curve is similar when the moxa-burning temperature is different.

#### 3.1.2. Influence of moxa stick radius on temperature distribution

**Fig 7:**
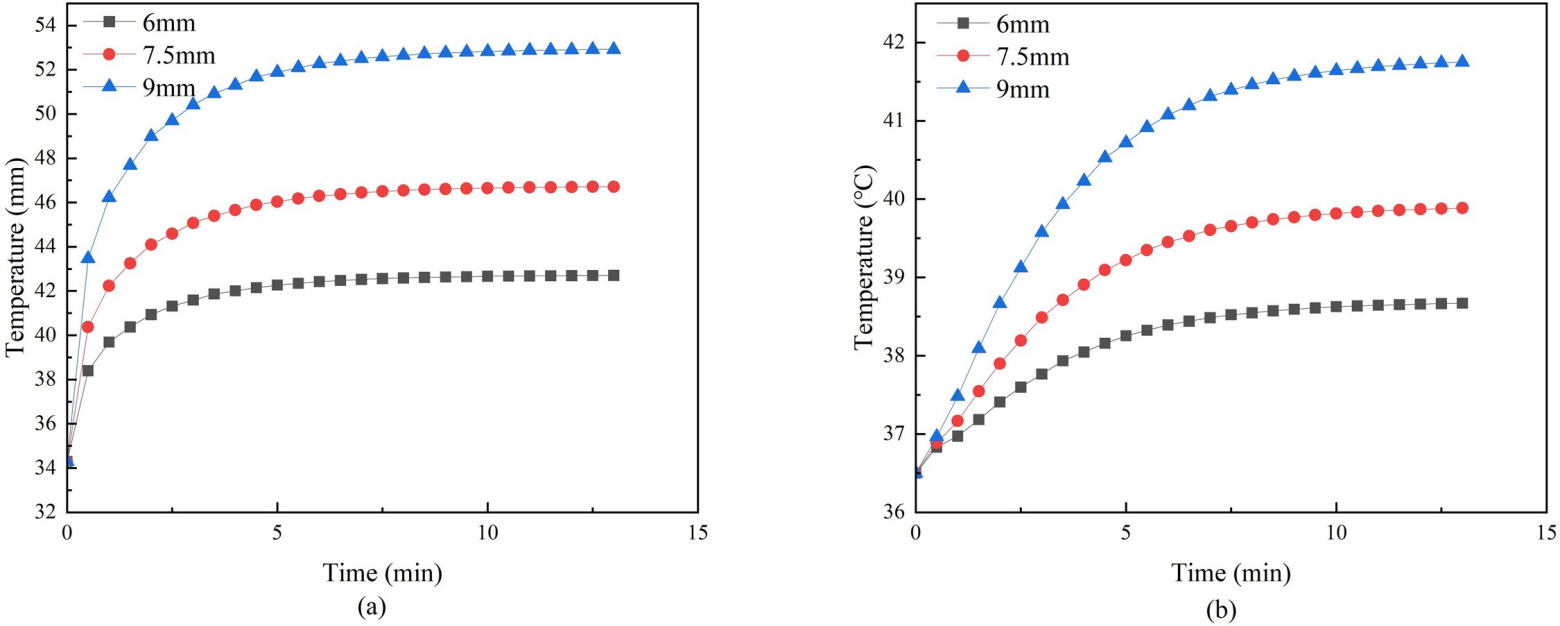
**(a)** Temperature curve at moxibustion point with different moxa radius. **(b)** Temperature curve at 5mm under moxibustion point with different moxa radius.

**Fig 8:**
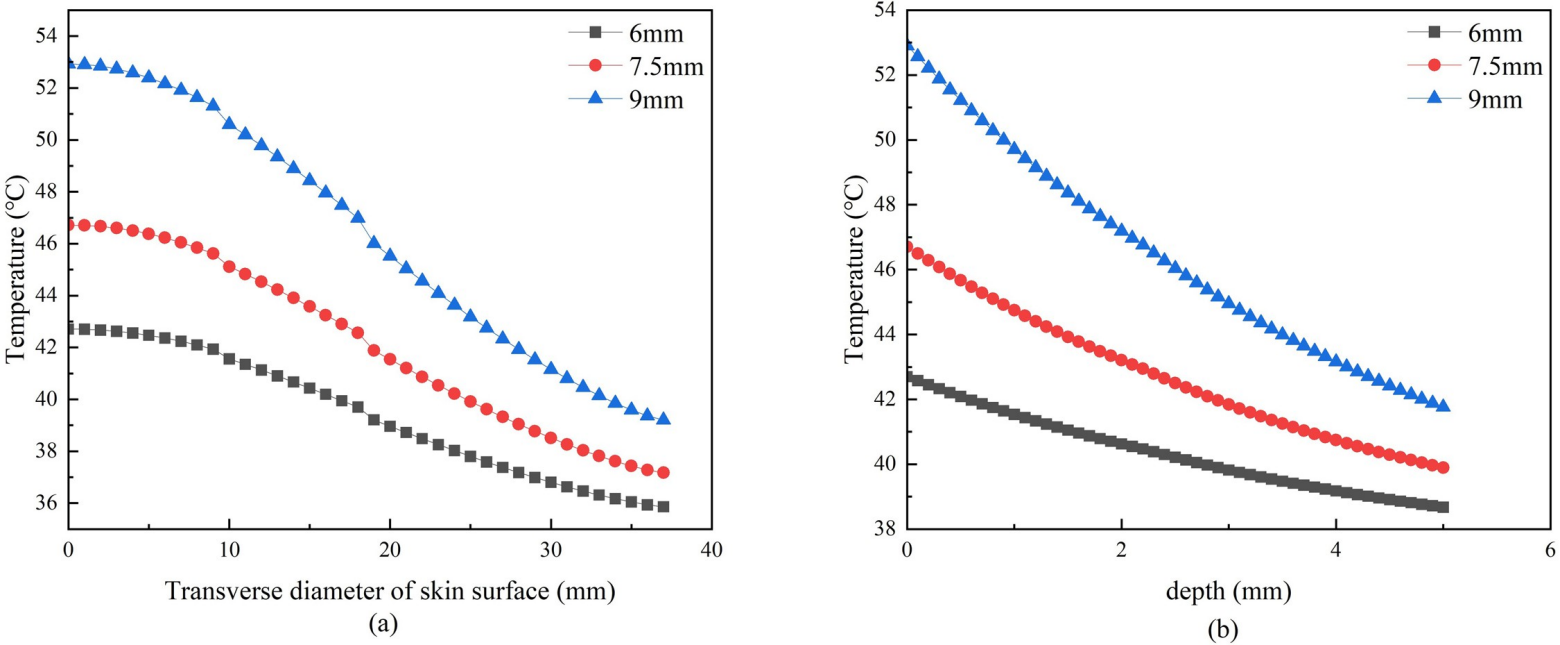
**(a)** Lateral temperature curves of skin surface under different moxa radii. **(b)** Vertical skin temperature curves under different moxa radii.

Under the conditions that the moxa-burning temperature, stick-to-skin distance, and skin moisture content were fixed, the moxa stick size was varied. Three radii of moxa sticks were set and simulated to obtain the temperature distribution on and inside the skin surface after mild moxibustion as shown in Figs 7 and 8.

Fig 7 shows that the moxa stick size has the most significant effect on skin temperature and impact the temperature rise rate. When the stick radius was 6mm, the skin surface temperature increased rapidly in the first 3 minutes, and when the radius was 7.5 or 9mm, the skin surface temperature increased rapidly in the first 5 minutes. At different radii, the temperature gradually reaches the maximum temperature. Fig 8 shows that the slopes of the lateral and vertical temperature attenuation curves are different when only the moxa stick size is changed. The larger the radius, the greater the slope of the temperature decay curve, and the faster the temperature decay.

#### 3.1.3. Effect of the stick-to-skin distance on temperature distribution

**Fig 9:**
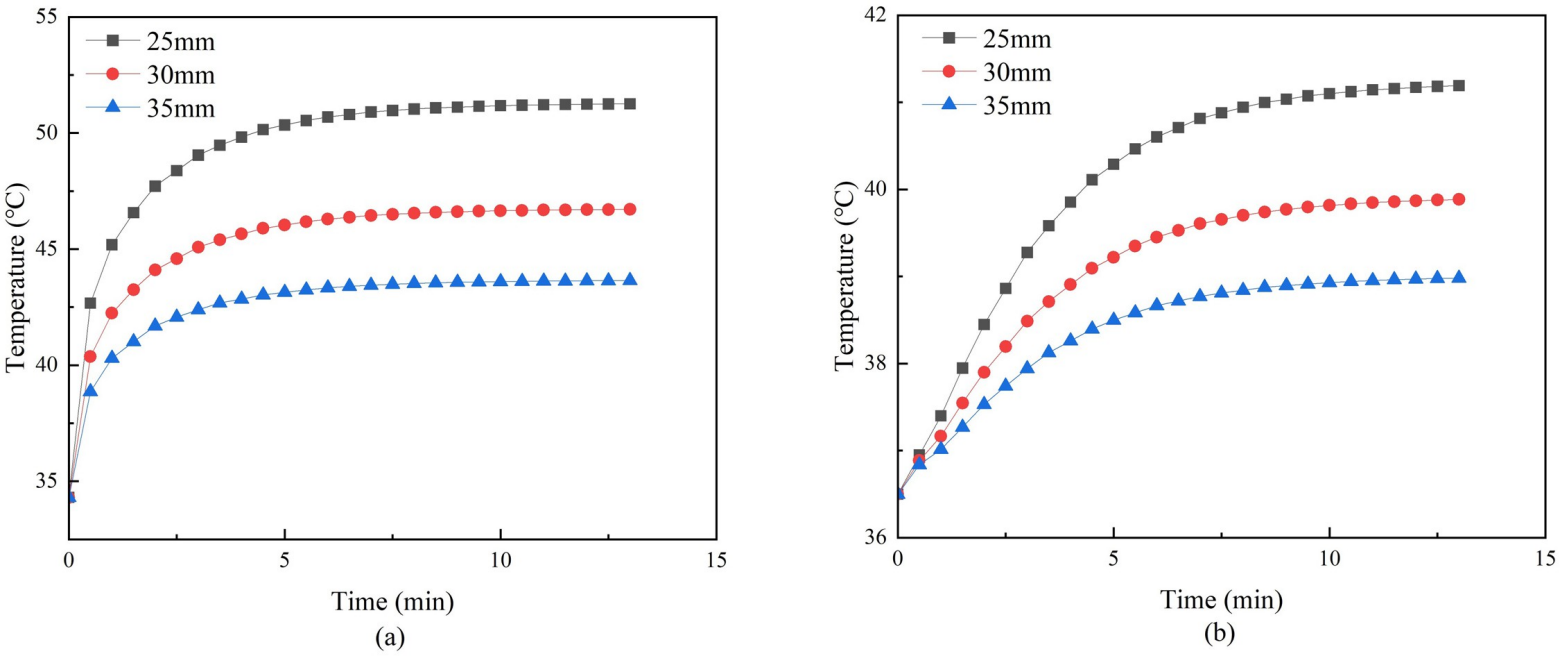
**(a)** Temperature curve of moxa point at different distances from the skin. **(b)** Temperature curve at 5mm under moxa point at different distances from the skin.

**Fig 10:**
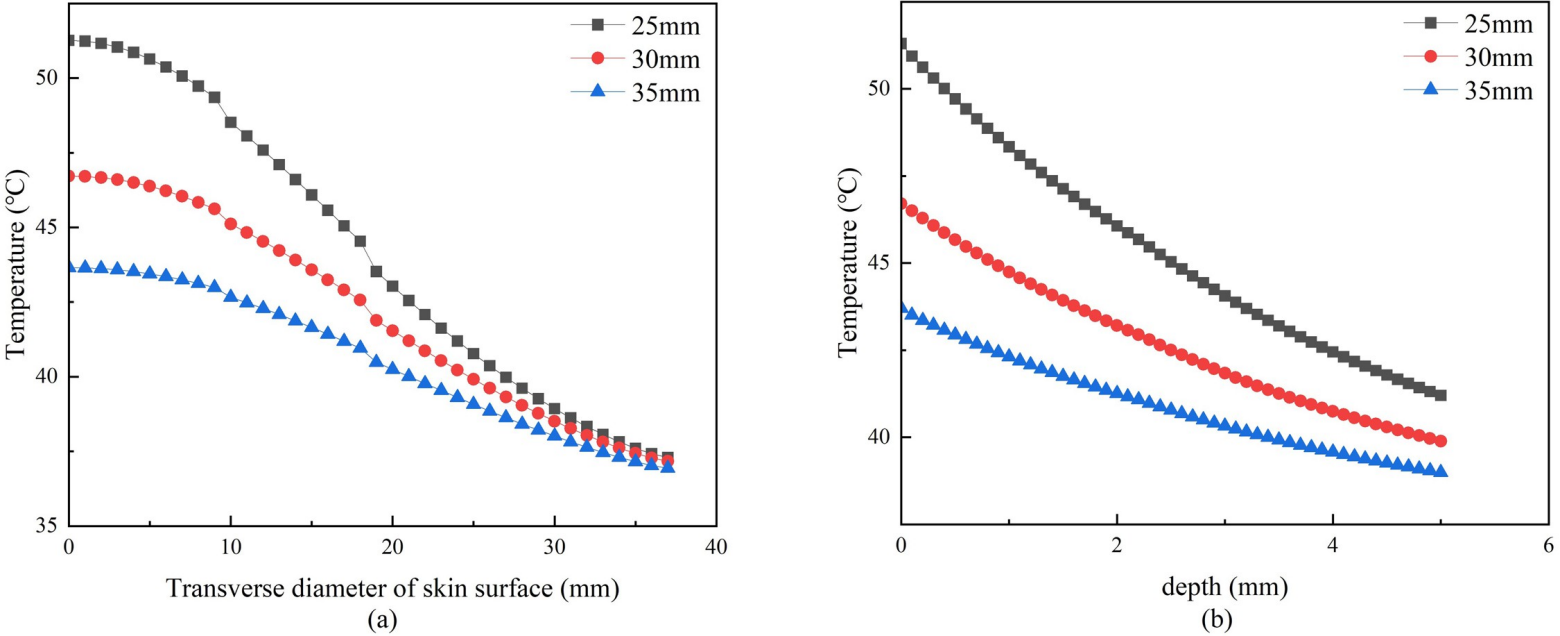
(a) Horizontal skin surface temperature curve at different distances from the skin. **(b)** Vertical skin temperature curve at different distances from the skin.

Under the condition that the mild moxibustion time, moxa-burning temperature, moxa stick size, and skin moisture content were unchanged, the stick-to-skin distance was changed. Three distances were set, and the simulation was conducted to obtain the temperature distribution on and inside the skin surface after mild moxibustion, as shown in Figs 9 and 10.

Fig 9 shows that the larger distance between the moxa stick and the skin, the lower the temperature on the skin surface and 5mm below the moxibustion point. During mild moxibustion, the skin surface temperature increases rapidly in the first 5 minutes, and gently in the 5–13 minutes. Next, the maximum temperature is gradually reached. Fig 10 shows that the smaller the distance between the moxa stick and the skin, the faster the skin lateral temperature decay. Fig 10A suggests that the temperature difference is not much when the skin surface is 30-40mm away from the moxibustion point. Besides as Fig 10B, the smaller the distance between the moxa stick and the skin, the slope of the temperature attenuation curve is higher.

#### 3.1.4. Effect of skin moisture content on temperature distribution

**Fig 11:**
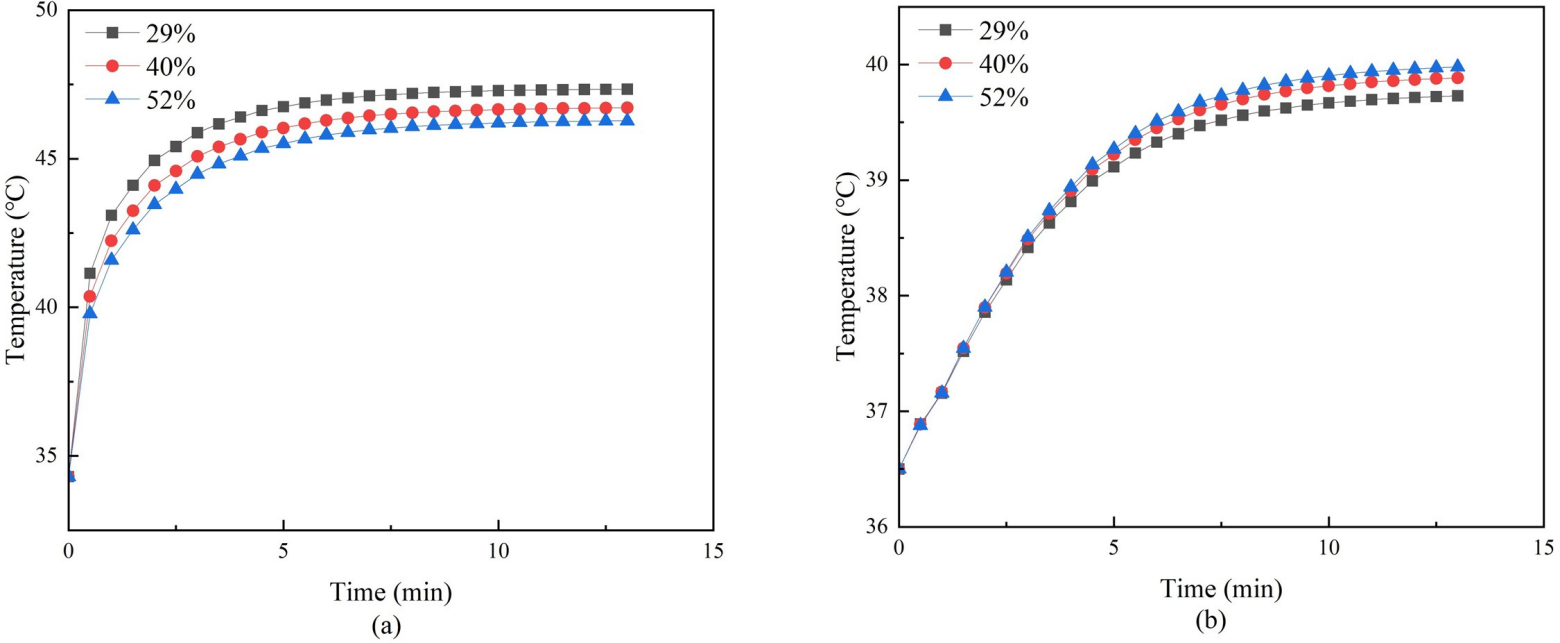
**(a)** Temperature curves of moxibustion points with different skin moisture content. **(b)** Temperature curve at 5mm under moxibustion point with different skin moisture content.

**Fig 12:**
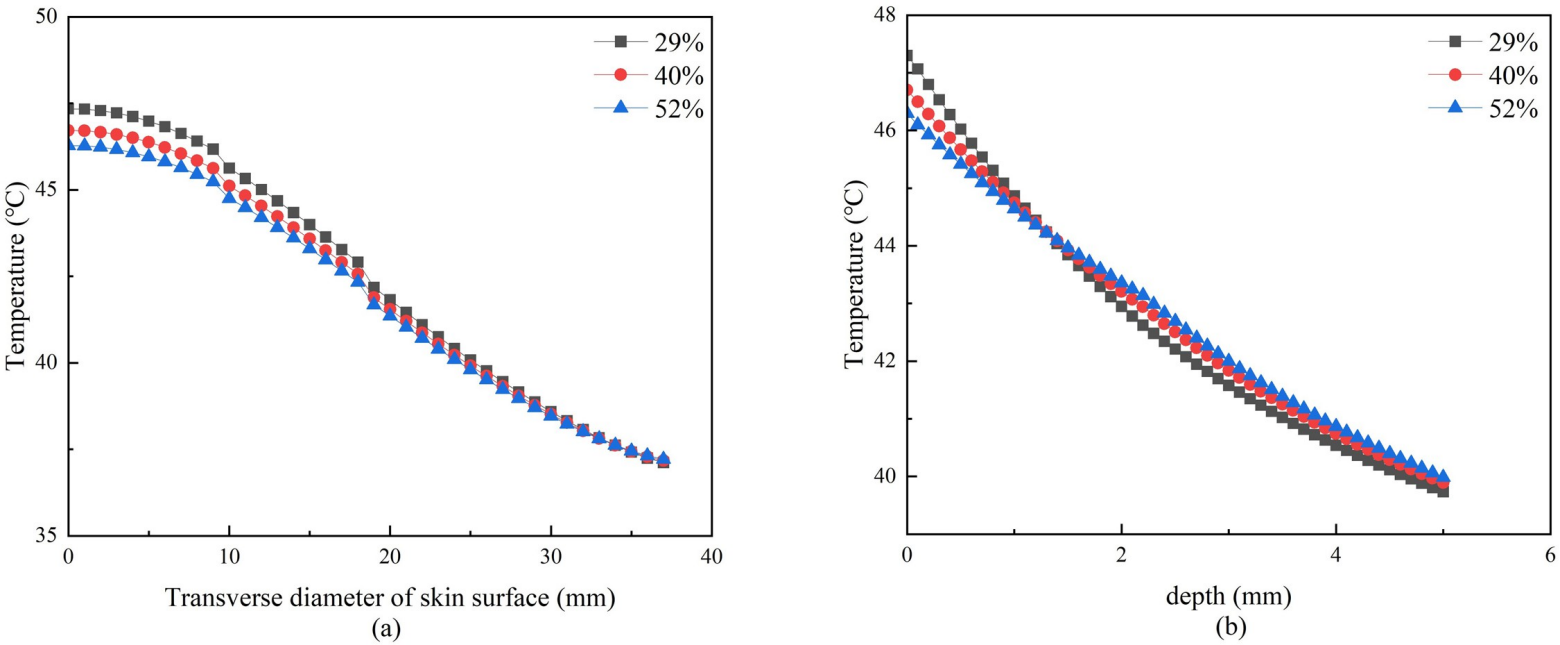
**(a)** Lateral temperature curve of skin surface with different skin moisture content. **(b)** Vertical skin temperature curve with different skin moisture content.

The skin moisture content was changed under the condition that the moxibustion time, moxa-burning temperature, moxa stick radius, and the stick-to-skin distance were unchanged. Three moisture content were set and simulated to obtain the temperature distribution on and inside the skin after mild moxibustion, as shown in Figs 11 and 12.

Fig 11 shows that the lower skin moisture content, the faster the temperature rise rate on the skin surface of the moxibustion point. But the worse the heat penetration, the slower the temperature rise rate of the 5mm under the moxibustion point. Fig 12 shows that the lower the moisture content, the faster the skin temperature decreases both laterally and vertically.

### 3.2. RBNN training

The error analysis results of the surrogate model are listed in Table 4.

**Table 4:** The error analysis results of the surrogate model.

option	RE (%)	*R* ^2^
HPM	[-1.356, 1.315]	0.993
ST	[-0.288, 0.589]	0.993

The results show that the RBNN-based response surfaces have high accuracy for the skin surface temperature and thermal penetration.

### 3.3. Uncertainty analysis of objective function

As listed in Table 5, the design domain and initial interval of moxibustion parameters include four influencing factors: the moxa-burning temperature (*t*_1_), the moxa stick size (*d*), the stick-to-skin distance (*h*),and the skin moisture content(*ω*_*c*_). The parameters affect the mild moxibustion efficacy to varying degrees.

**Table 5:** The value range and interval radius of moxibustion parameters.

Parameter name	Symbol	Value range	radius
Burning temperature of moxa stick (°C)	*t*_1_	[600,650]	2.5
Moxa stick thickness(mm)	*d*	[12,18]	0.15
Distance between moxa stick and skin(mm)	*h*	[25,35]	0.8
the skin moisture content	*ω_c_*	[29,52]	1.1

As listed in Table 6, the sequential quadratic programming is used to calculate the response intervals of the moxibustion indicators based on the created RBNN model.

**Table 6:** The objective function value interval of the mild moxibustion.

Index parameters	General value	Value range
HPM (°C)	38.5	[37.5,43.5]
ST (°C)	45.5	[39.5,58.1]

### 3.4. Solution of the nonlinear interval optimization

By introducing RPDI, the classical optimization algorithms can be used to solve the uncertain interval optimization. The study calculated the optimal solutions under different RPDIs.

The results show that when *λ* is more than 1, the upper limit of the skin surface temperature interval is less than the allowable value. When the RPDI is 0.8 or 0.9, the upper limit of the surface temperature interval exceeds the allowable value (45.5 °C).

## 4. Discussion

### 4.1. Single factor analysis of operating parameters

When the moxa-burning temperature is different, Fig 5 shows that the higher the moxa-burning temperature, the higher the skin surface temperature and the temperature at 5mm under the moxibustion point. There is a positive correlation. And the higher the moxa-burning temperature, the faster the temperature of this two places rise rate. Fig 6 showed that the slope of the temperature curve is similar when the moxa-burning temperature is different. It indicated that the moxa-burning temperature will not affect the heat conduction rate of the skin.

When the moxa stick radius is different, Fig 7 shows that the moxa stick size has the most significant effect on skin temperature and impact the temperature rise rate. Fig 8 shows that the slopes of the lateral and vertical temperature attenuation curves are different when only the moxa stick size is changed. The larger the radius, the greater the slope of the temperature decay curve, and the faster the temperature decay. It indicates that the thermal conductivity of the skin and fat will greatly hinder the temperature propagation.

When the stick-to-skin distance is different, Fig 9 shows that the stick-to-skin distance is inversely correlated with skin temperature. Fig 10 shows that the stick-to-skin distance has little effect on the temperature of the skin surface at 30-40mm from the moxibustion point.

When the skin moisture content is different, Fig 11 shows that the lower skin moisture content, the faster the temperature rise rate on the skin surface of the moxibustion point. But the worse the heat penetration, the slower the temperature rise rate of the 5mm under the moxibustion point. Fig 12 shows that the lower the moisture content, the faster the skin temperature decreases both laterally and vertically.

Through the above single factor analysis, it can be clearly seen that the changes of the moxa-burning temperature, moxa stick radius, stick-to-skin distance, and skin moisture content have obvious effects on skin surface temperature *ST* and skin heat penetration *HPM*. Thus the four parameters were selected as optimization parameters to study their influences on the moxibustion efficacy.

### 4.2. Uncertainty analysis of objective function

**Fig 13:**
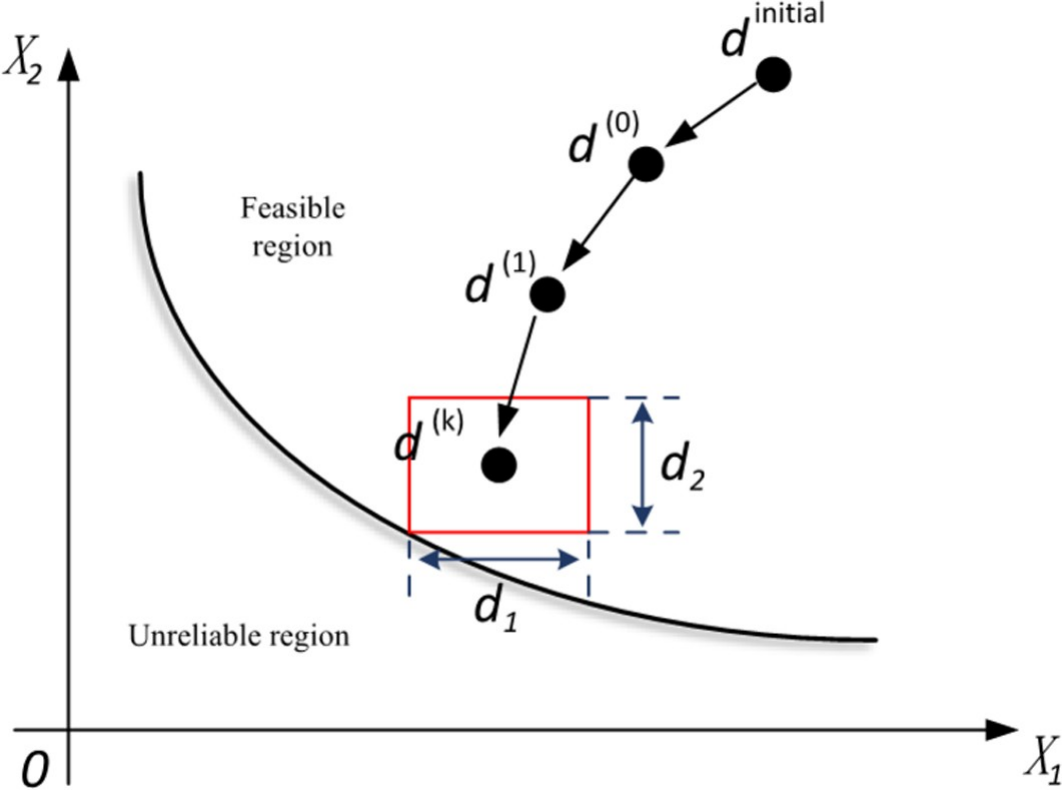
The interval uncertainty optimization principle.

When the parameter uncertainty is ignored, the obtained thermal penetration reaches the target value, and the mild moxibustion efficacy achieves the expected effect. But under the influence of parameter uncertainty, the value of heat penetration is unreliable because the skin surface temperature may exceed 45.5 °C. In other words, the uncertainty of moxibustion parameters leads to large fluctuations in thermal penetration, so the moxibustion efficacy fails to achieve the expected effect. The moxibustion indicator value falls in a change region, not a point. As shown in Fig 13, the part of the area where the moxibustion index value changes are located in the unreliable region, indicating that the effect of moxibustion is unreliable. The uncertainties are quantified as interval variables, whereby the parameter uncertainty domain forms a multidimensional box.

To obtain a reliable design, it should be ensured that the moxibustion indicator variation domain is entirely within the feasible domain. Therefore, the interval uncertainty optimization method can ensure the reliability of the design solution and maximize its efficacy.

### 4.3. Solution of the nonlinear interval optimization

The results show that when *λ* is more than 1, the upper limit of the skin surface temperature interval is less than the allowable value. It suggests that the temperature response interval meets the requirements, the mild moxibustion treatment is in a reliable operating condition. Compared to the temperature values obtained by the deterministic optimization, the upper limit of thermal penetration is significantly increased, enhancing the efficacy.

**Fig 14:**
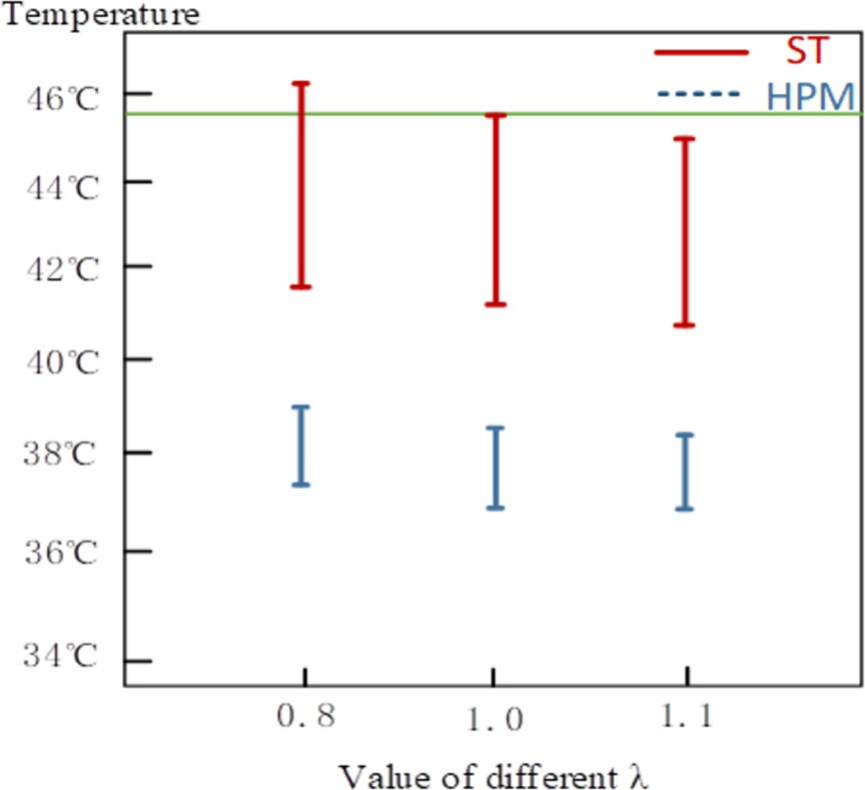
The optimal solutions under different RPDIs.

The effect of RPDI on the optimal solution of thermal penetration and surface temperature is investigated, and the interval optimal solutions under different RPDIs are discussed. The results are visually compared as shown in Table 7 and Fig 14. The increase in RPDI decreased the values of thermal penetration and surface temperature, indicating that increasing the moxibustion reliability reduces efficacy. When the RPDI is greater than or equal to 1, the surface temperature interval is entirely within the allowable temperature range. Thus, the moxibustion treatment is reliable. When the RPDI is 0.8 or 0.9, the upper limit of the surface temperature interval exceeds the allowable value (45.5 °C), indicating that the patient may feel uncomfortable during mild moxibustion.

**Table 7:** The optimal solutions under different RPDIs.

*λ* value	0.8	0.9	1.0	1.1
ST(°C)	[41.81,46.24]	[41.63,45.90]	[40.99,45.50]	[40.59,44.98]
HPM(°C)	[37.59,38.84]	[37.44,39.63]	[37.35,38.60]	[37.27,38.51]

## 5. Conclusions

The traditional mild moxibustion treatment studies rarely considered the moxibustion parameter uncertainties, resulting in the failure to maximize the efficacy. To accurately measure the influence of parameter uncertainties, the interval uncertainty analysis and optimization for the mild moxibustion were implemented to ensure the moxibustion reliability and efficacy. Firstly, the mild moxibustion simulation model was established, and the corresponding indicator parameters were determined. Secondly, a single factor analysis was conducted after setting the operating parameters, and the constraint and objective functions were created based on a surrogate model, using the RBNN regression method. Thirdly, the interval uncertainty analysis method was introduced to obtain the reliability analysis results of moxibustion. The results showed that the temperature values under deterministic moxibustion conditions were reliable, but the moxibustion parameter uncertainties might lead to unreliable moxibustion conditions. Later, a nonlinear interval optimization model was formulated. The RPDI was applied to transform the nonlinear interval optimization model into deterministic optimization. The genetic algorithm was employed to solve the deterministic optimization problem. The results show that the obtained optimal solution significantly improved the efficacy with meeting the reliability requirements of moxibustion temperature. Finally, the effect of RPDI on the optimal solution was investigated. The results showed that the increase in RPDI decreased the values of thermal penetration and surface temperature. In other words, increasing the moxibustion reliability reduced efficacy. Therefore, the value of RPDI should be given according to the actual application for achieving optimal efficacy.

## Supporting information

S1 Dataset Single factor analysis. This is the data from our single-factor analysis of the four operational parameters.
(XLSX)

S2 Dataset Latin sampling. This is the simulation data and the fitting data obtained according to the Latin sampling results. We also process the data generated by simulation and fitting.
(XLSX)
